# Assessment of Community Awareness and Screening of Chagas Disease in the Latin American Community of Greater New Orleans

**DOI:** 10.3390/tropicalmed8120515

**Published:** 2023-12-05

**Authors:** Claudia Herrera, Kerlly J. Bernabé, Eric Dumonteil, James DeCuir, Julie M. Thompson, Mariana Avendano, Weihong Tu, Maxwell M. Leonhardt, Bianka A. Northland, Jynx Frederick, Bryn Prieto, Angel Paternina-Caicedo, Emma Ortega, Maria Fonseca, Marcela Hincapie, Margarita Echeverri

**Affiliations:** 1 Department of Tropical Medicine, Tulane University School of Public Health & Tropical Medicine, New Orleans, LA 70112, USA; kbernabe@tulane.edu (K.J.B.); edumonte@tulane.edu (E.D.); jdecuir2@tulane.edu (J.D.); jthompson4@tulane.edu (J.M.T.); mavendano@tulane.edu (M.A.); wtu1@tulane.edu (W.T.); jfrederick@tulane.edu (J.F.); 2 Department of Medicine, Tulane University School of Medicine, New Orleans, LA 70112, USA; mleonhardt@tulane.edu (M.M.L.); bnorthla@tulane.edu (B.A.N.); 3 Department of Social, Behavioral, and Population Sciences, Tulane University School of Public Health & Tropical Medicine, New Orleans, LA 70112, USA; bprieto@tulane.edu; 4 Department of Epidemiology, Tulane University School of Public Health & Tropical Medicine, New Orleans, LA 70112, USA; apaterninacaicedo@tulane.edu; 5 Office of Public Health-Infectious Disease Epidemiology, Louisiana Department of Health, New Orleans, LA 70802, USA; emma.ortega@la.gov; 6 College of Pharmacy, Xavier University of Louisiana, New Orleans, LA 70125, USA; mfonseca@xula.edu (M.F.); mhincapi@xula.edu (M.H.); mechever@xula.edu (M.E.)

**Keywords:** *Trypanosoma cruzi*, diagnostic, surveillance, screening, Chagas disease barriers, Latin American community, education

## Abstract

Chagas disease is a public health problem in the Americas, from the southern United States (USA) to Argentina. In the USA, less than 1% of domestic cases have been identified and less than 0.3% of total cases have received treatment. Little is known about affected immigrant Latin American communities. A prospective study was conducted to assess knowledge about Chagas disease among the Latin American community living in the Greater New Orleans area. Participants answered a baseline questionnaire, viewed a short educational video presentation, completed a post-presentation questionnaire, and were screened with an FDA-approved blood rapid diagnostic test (RDT). A total of 154 participants from 18 Latin American countries (*n* = 138) and the USA (*n* = 16) were enrolled and screened for *Trypanosoma cruzi* infection. At baseline, 57% of the participants knew that Chagas disease is transmitted through an insect vector, and 26% recognized images of the vector. Following the administration of an educational intervention, the participants’ knowledge regarding vector transmission increased to 91% and 35% of participants were able to successfully identify images of the vector. Five participants screened positive for *T. cruzi* infection, indicating a 3.24% [95%CI: 1.1–7.5%] prevalence of *Trypanosoma cruzi* infection within the Latin American community of the New Orleans area. Results highlight the urgent need for improving access to education and diagnostics of Chagas disease.

## 1. Introduction

Chagas disease is a public health problem in the Americas, from the southern United States of America (USA) to Argentina [1]. The disease is caused by an infection with the protozoan parasite *Trypanosoma cruzi*. Approximately 6–8 million people are known to be infected in endemic countries, and about 65–100 million additional people remain at risk for infection in these areas [2]. The parasite can be transmitted by triatomine insects, vertical transmission, blood donations, organ transplants, and through consumption of food or drink contaminated by triatomine feces [3]

Estimates for the number of individuals living in the USA affected by Chagas disease vary by study; however, an overall estimate suggests there are approximately 300,000 people living with the condition in the USA, positioning the USA as the sixth most burdened country in terms of global disease burden from Chagas disease [1,4]. Despite this disease burden, less than 1% of domestic cases have been identified and less than 0.3% have been treated, increasing the risk for disease progression to cardiac or digestive forms [5]. In 2022, Irish et al. estimated that there are 33,231 individuals infected with *T. cruzi* living in the state of Texas and 15,586 living in the state of Florida, where there is no mandatory reporting [6]. Additionally, Irish et al. estimated that there were 57,000 patients living with Chagas cardiomyopathy, an estimate nearly 1.5 times greater than previously reported estimates [6]. Another analysis reported that 27.7% of hospitalized patients in the USA with a primary or coexisting diagnosis of Chagas heart disease were from the Southern USA [7]. Notably, these figures are only rough estimates. Chagas disease remains underreported, and individuals living with the disease experience systemic, structural, clinical, and psychosocial barriers to the effective prevention, detection, and management of the disease [8].

Among the primary barriers are a lack of awareness of the disease among both patients and physicians, limited testing options, and limited access to medications [9,10]. There is limited research describing community awareness and knowledge of Chagas disease in the USA [6,11,12]. Additionally, knowledge of Chagas disease prevalence remains insufficient due to the absence of awareness and systematic screening. This gap persists, even in states like Louisiana, where reporting is mandatory. In fact, reporting of Chagas disease is mandatory in only seven states: Arizona; Arkansas; Louisiana; Massachusetts; Mississippi; Tennessee; and Texas [13,14]. Additional research in the southern USA is needed to gain a more comprehensive understanding of the Chagas disease risk landscape. This insight is crucial for crafting effective interventions aimed at educating communities regarding their susceptibility to the disease. In Louisiana, the 2020 Hispanic population was reported to be 322,549 individuals [15]; among this population, there were twelve cases of Chagas disease reported (including seven suspected to be autochthonous) [16,17]. The present study implemented an educational intervention and screened for *T. cruzi* infection in a sample of Latin Americans living in the Greater New Orleans area of Louisiana.

## 2. Materials and Methods

### 2.1. Study Population

Participants were recruited between 2021 and 2023 during three health fairs held in New Orleans at the Hispanic Apostolate (Archdiocese of New Orleans). Eligible participants were adults 18 years and older residing in the Greater New Orleans area who self-identified as Latin American. This included both English and Spanish speakers, encompassing first and second-generation individuals. The first Chagas disease fair was held over three days from 30 April to 2 May 2021. The second and third fairs were single-day events, taking place on 5 November 2022 and 15 April 2023.

English and Spanish-language flyers advertising the events were distributed by community leaders to businesses, organizations, and centers serving the Latin American community. In addition, the events were advertised by two local Spanish-speaking radio stations and a TV news network. Interested individuals could pre-register to participate in the study, and same-day registration was also offered onsite.

Written informed consent and the Health Insurance Portability and Accountability Act (HIPAA) authorization in Spanish or English, based on the preference of the participant, were obtained from all enrolled participants. Participants were not asked about their immigration status and were assigned a unique study identification number to protect their privacy. This study was approved by the Tulane University Institutional Review Board (Study No. 2020-169) and Xavier Institutional Review Board (Study No. 796).

### 2.2. Surveys and Educational Intervention about Knowledge Regarding Chagas Disease

Participants completed an online or paper questionnaire (see Supplementary Materials) in English or Spanish. At baseline, information on age, gender (female, male, other, and prefer not to answer), country of birth, and length of time living in the USA was collected. Participants answered multiple-choice questions about the causative agent of Chagas disease and its mode of transmission (four answer options including I do not know/I’m not sure). Participants were also asked if they had ever seen triatomine insects, kissing bugs, vampire bugs, or conenose bugs. Two questions asked participants to identify images of the triatomine vectors that transmit *T. cruzi*. Images of triatomines were taken and modified from Behrens-Bradley et al. [18]. Participants were also asked questions about their exposure and interest regarding the disease: (a) if they had been diagnosed with Chagas disease by a physician; (b) if they would like to learn more about Chagas disease; (c) if they would like to know if they have a *T. cruzi* infection; and (d) if they wanted to get tested for a *T. cruzi* infection.

After completing the baseline survey, participants were shown a 12 min educational video (the intervention) on Chagas disease, transmission, screening, treatment, and mandatory reporting of positive test results to the Louisiana Department of Health (LDH). The video presentation, led by a Chagas disease expert from the team, was delivered in English or Spanish, per the preference of the participant.

The post-intervention survey retained the original knowledge-based questions from the initial assessment, and it included two additional questions: one gauging the clarity of the video presentation and another asking participants if they intended to talk with their friends and family about Chagas disease.

### 2.3. Data Collection

Survey responses were collected and stored using Qualtrics (Qualtrics, Provo, UT, USA). For purposes of data analysis, survey responses that were I don’t know/I’m not sure were labeled as incorrect answers. Responses of I’m not sure to the question in which participants recalled seeing a triatomine were labeled no. Participants who successfully identified at least one of three images of triatomines were considered to have correctly recognized triatomine insects.

Data was stratified based on the estimated prevalence of *T. cruzi* infection in individual countries, dividing them into high and low infection categories of prevalence (>1% vs. <1%). Countries with >1% prevalence include Bolivia (6.1%), Argentina (3.6%), Ecuador (1.3%), El Salvador (1.2%), and Guatemala (1.2%) [1]. Latin American countries with <1% prevalence include Honduras (0.9%), Colombia (0.9%), Mexico (0.7%), Chile (0.6%), Nicaragua (0.5%), Venezuela (0.7%), Panama (0.5%), Peru (0.4%), Uruguay (0.2%), and Brazil (<0.1%), and the USA (estimated <1%) [1]. In addition, survey responses were stratified by whether the participant’s country of birth had a national control program (yes or no). Countries with national control programs include Argentina, Bolivia, Brazil, Chile, Colombia, Ecuador, Guatemala, Mexico, Honduras, Nicaragua, Uruguay, and Venezuela. Countries that do not have national programs are El Salvador, Panama, Peru, and the USA. The Caribbean islands are not considered endemic regions and do not have control programs; there is no prevalence data reported on Cuba, Puerto Rico, and the Dominican Republic. Thus, survey responses for participants from these countries were only stratified by country of birth. 

### 2.4. Chagas Disease Screening

A USA Food and Drug Administration (FDA)-approved blood rapid diagnostic test (RDT) (Chagas Detect^TM^ plus) was performed on participants who consented to the screening. The results of the RDT were recorded and then documented as digital photos. Following HIPAA regulations, participants were individually notified of their test results in a private room. Participants who tested positive completed the LDH Confidential Disease Case Report Form and returned it to the onsite primary investigator. Participants were informed that in the event of positive RDT results, the test would be confirmed by the USA Centers for Disease Control and Prevention (CDC). An onsite infectious disease physician from Tulane University School of Medicine provided information to those participants who tested positive on how to request an appointment for diagnostic confirmation in a reference laboratory, as well as information on clinical follow-up and potential treatments, if needed. Medical students were also onsite to provide participants with additional information. Participants with negative results were informed about their results, and all participants were encouraged to share their knowledge of Chagas disease with others.

### 2.5. Statistical Analysis

Categorical variables were described using percentages, while continuous variables were reported as median, with their interquartile ranges. Responses to knowledge questions were reported separately before and after the intervention and compared by gender, age groups (<50 years old, ≥50 years old), and by the prevalence level of *T. cruzi* infection and existence of national Chagas disease control program in the country of birth. McNemar’s chi-square test was used to compare the proportions of correct responses before and after the intervention for each knowledge question. A *p*-value < 0.05 was considered statistically significant. All analyses were conducted with the R statistical package (v3.2.1) [19].

## 3. Results

### 3.1. Study Population

A total of 154 participants were enrolled and screened for *T. cruzi* infection (64 in 2021, 40 in 2022, 50 in 2023). The sample included individuals from the USA and 18 countries in Latin America, including three Caribbean countries (Table 1). Most of the participants were female (*n* = 103, 67%), born in Central America (*n* = 85, 53 female), and completed surveys in Spanish (*n* = 129, 84%).

The median age was 52 years (IQR: 40–63) and the median time living in the USA was 18 years (IQR: 8–25). This study had participants who recently moved to the USA (2 days) and participants who have lived in the USA for as long as 69 years. Eighty-one percent (124/154) of participants were born in countries with a low prevalence of disease (<1%), 16% (25/154) from countries with a high prevalence (>1%), and 3% (5/154) were from countries without reported data. Eighty-one percent (124/154) of participants were born in countries with a national Chagas disease control program.

### 3.2. Awareness and Knowledge of Chagas Disease at Baseline and after Educational Intervention

At baseline (pre-educational intervention; *n* = 154), 57% of participants answered correctly on the causative agent and vector of Chagas disease, 4% answered incorrectly, and 39% were unsure (Table 2). Over half had seen triatomines, 31% answered no, and 16% were unsure. About a quarter recognized at least one of the images of triatomines at baseline, indicating inadequate knowledge of disease vectors.

Knowledge of the causative agent and vector of Chagas disease improved from 57% at baseline to 91% at post-assessment (*n* = 147) (*p*-value < 0.05) (Table 2). Recognition of triatomine insects improved significantly (*p* < 0.05) from 26% at baseline to 35% at post-assessment. This result indicates an increase in knowledge but also highlights a knowledge gap in disease vectors; while at baseline, 52% reported having previously seen triatomines, the percentage increased to 65% at post-assessment. There were no statistically significant differences in knowledge by gender, age, prevalence in country of birth, and existence of a national program.

**Figure 1:**
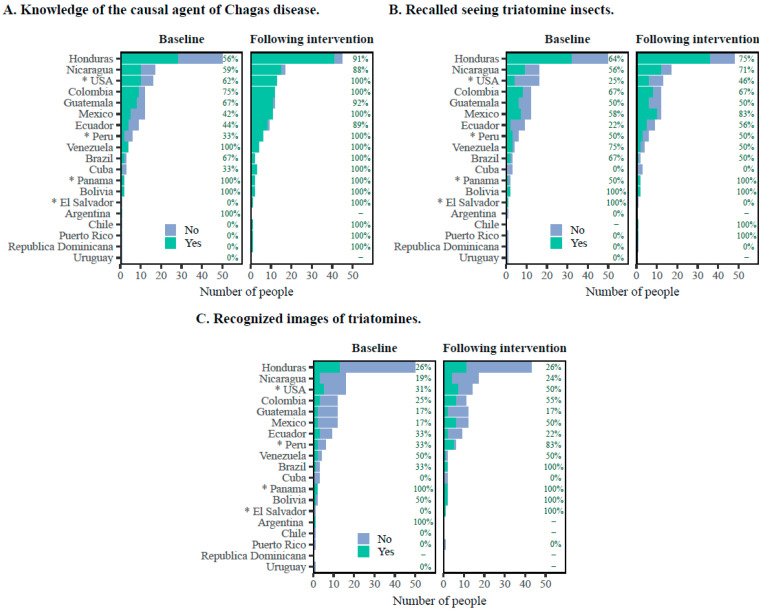
Participant responses to survey questions at baseline and following educational intervention by country of birth. (**A**) General knowledge about Chagas disease. (**B**) Recalled seeing triatomine insects. (**C**) Recognized at least one image of a triatomine. Data are presented as the number of participants and percentage. * Countries that have never had a national program for the control of Chagas disease.

Thirty-two percent of participants were individuals born in Honduras (Figure 1), of which 56% answered correctly on the causative agent of Chagas disease at baseline. This percentage increased significantly (*p* < 0.05), to 91% post assessment (Figure 1). 

Participants’ willingness to learn more about Chagas disease remained consistent from baseline (131 out of 153, 86%) to the post-assessment (119 out of 146, 82%). Interest in knowing their Chagas disease status was also consistent with baseline (147 out of 154, 95%, responded yes) and at post-assessment (144 out of 146, 99%, responded yes). The willingness of participants to get tested for *T. cruzi* infection also remained consistent from baseline 151 out of 154, and 98 (149 out of 154, 98%, responded yes) to post-assessment (145 out of 146, 99%, responded yes). For the two additional post-assessment questions, most participants reported that the presentation was clear (144 out of 146, 99%, responded yes) and that they would talk to others about Chagas disease and about the study (142 out of 146, 97%, responded yes). 

### 3.3. Chagas Disease Screening

Most participants (99%, 153/154) at baseline responded that a doctor had not told them they had Chagas disease, and one participant answered that they had been told they had Chagas disease. Five participants (3.24%, [95%CI: 1.1–7.5%]) screened positive for *T. cruzi* infection, including one participant who had previously been diagnosed with Chagas disease. Four of these participants were >50 years old, and two were male. While none of the infected were born in the USA (one was born in Bolivia, three in Honduras, and one in El Salvador), two (40%) individuals had been living for more than 20 years in the USA (Supplementary Table S1). All the participants were notified to the LDH for diagnostic confirmation and a list of infectious disease doctors was provided for follow-up.

## 4. Discussion

This study evaluated the impact of Chagas disease education among a sample of the Latin American community of Greater New Orleans, revealing a high interest in being tested for the disease. Considering the scarcity of prevalence studies in the USA, this community-based screening study highlights the probable significant presence of Chagas disease in the Greater New Orleans area, with 3.24% [95%Cl: 1.1–7.5%] screening positive in a sample of 154 Latin Americans. The majority of these participants reside in Orleans Parish, making up around 15% of Louisiana’s Latin American population of 322,549 [15], primarily comprising individuals from Honduras.

These screening test results must be confirmed with another FDA-approved test, due to variable test performance among populations [20,21]. Nonetheless, even using a low estimate for the true prevalence of infection of 1%, there may be over 3000 cases not yet diagnosed or referred to treatment in Louisiana [10,22]. Therefore, prioritizing testing within affected communities is crucial for quantifying Louisiana’s disease burden. Community-level screening initiatives can effectively link identified cases to necessary clinical care. 

Such an initiative would yield accurate prevalence data for Chagas disease, crucial in identifying the risk of developing heart disease and other chronic manifestations of Chagas disease among vulnerable populations.

Improved screening for *T. cruzi* is needed in the USA, particularly among Latin American immigrant communities. Considering the increased migration of Latin American populations across the globe and challenges with health access and equity, educating this population about Chagas disease and how they can access health services provides the community with effective tools to overcome barriers to testing and access to treatment due to stigmatization [23].

Several studies have highlighted the necessity of connecting education efforts with Chagas disease prevention. Information, education, and communication (IEC) have been formally included as part of some Chagas disease programs in Latin American countries as a strategic, essential, and complementary axis for the success of the implementation of Chagas disease programs in the community [24,25]. Diverse approaches to IEC, addressing the different dimensions of the disease, are described in a recent review [26]. Nevertheless, there is a limited body of research on community awareness and knowledge of Chagas disease. Our results show that 57.3% of Latin American participants had seen triatomines before, indicating a general awareness of triatomines and the ability to recognize them. A similar study conducted in Los Angeles, California, reported that 62% of their study population had seen triatomine vectors [11]. Our New Orleans-based study population included 32.7% of participants born in Honduras. Among them, twenty-eight (56%) knew the cause of Chagas disease at baseline. This aligns with findings from a study conducted in a rural Honduran community, where 74% to 81% of participants learned how Chagas disease was transmitted [27]. Our study revealed an overall positive impact from the educational intervention, notably increasing knowledge about the human transmission of Chagas disease. These results underscore the significant role of disease education in enhancing community awareness and knowledge.

Importantly, most of the participants whose countries of origin have a national Chagas program exhibited greater prior knowledge of Chagas disease than those coming from countries without a national control program, demonstrating the positive impact that these programs have on the population-level knowledge about Chagas disease. Specifically, our study included 82 individuals (53%) born in Central America (Table 1), where Chagas disease programs have been carried out for several years [6,14]. It is noteworthy that 59% (48/82) of this population knew that Chagas disease is caused by a parasite and is transmitted by an insect vector at baseline. When stratified by country of birth, all participants from Mexico, Guatemala, Ecuador, Colombia, El Salvador, and Peru correctly answered the question regarding the transmission of Chagas disease. In addition, participants from across the USA and Mexico, Central America, and South America could recall whether they had seen a triatomine. Moreover, the percentage of participants who were able to correctly identify an image of a triatomine increased from 26.9% at baseline to 53.8% after the intervention; participants born in the USA, Colombia, and Honduras exhibited more significant increases compared to participants born in Ecuador. 

Although the United States was initially considered a non-endemic area, it has a different epidemiological setting than non-endemic countries [4], with a well-described enzootic *T. cruzi* transmission in the southern region, involving 11 triatomine species identified in 29 out of 50 states [28,29], and a range of mammalian hosts [30]. There is an increasing number of autochthonous and congenital transmission cases reported [31], and the USA was ranked number seven in terms of the number of individuals with Chagas disease in the Western Hemisphere [30]. If not detected and treated in time, individuals with Chagas disease risk developing heart disease and other serious complications. Despite the number of estimated cases of Chagas disease in the USA, no effort to conduct widespread surveillance is performed in states considered at the highest risk of *T. cruzi* infection [14].

This study has strengths and limitations. First, the use of survey instruments and educational training in both English and Spanish is an important strength. As most participants preferred Spanish over English, the bilingualism of this study likely allowed it to reach individuals with limited English proficiency who may otherwise be underserved or excluded in research and healthcare programs. Second, this study used plain language and regional terms in Spanish to accommodate different literacy levels and ascertain whether participants had seen vectors. Given that different countries in Latin America use distinct terms for vectors, it was imperative to incorporate these terms into the training and surveys. This ensures that participant responses reflect their knowledge of the vectors. On the other hand, in terms of limitations, there could be some bias introduced by the recruitment method used since it was conducted only in one institution. Non-Latin American individuals in the Greater New Orleans area could also be at risk of Chagas disease due to the possibility of autochthonous transmission and blood product transmission. Additionally, the small sample size can limit the generalizability of the various subgroup analyses, especially for the country-specific analyses. Nevertheless, our overall conclusions regarding the benefits of educational programs and the benefits of increased screening hold true and indicate the need for future studies with larger sample sizes, more expansive recruitment procedures, and educational programs of greater scope. Furthermore, the study did not collect information on other forms of transmission (i.e., congenital, blood transfusion) to further understand community knowledge and risk. Finally, this study reported screening for *T. cruzi* using a single RDT, and confirmatory testing is still needed for a definitive diagnosis. 

## 5. Conclusions

There is an important prevalence rate of *T. cruzi* infection among immigrant Latin American residents within the Greater New Orleans area, demonstrating the need to improve screening for Chagas disease to confirm cases in this population and provide timely treatment and referral to proper care. It is of the utmost importance to provide the community with correct information about the disease and emphasize that it is not a terminal illness, that there is a treatment, and that timely diagnosis and treatment can prevent the progression of the disease to possible severe forms such as Chagasic cardiomyopathy, as well as avoid vertical transmission.

## Figures and Tables

**Table 1:** Sociodemographic characteristics of study participants, Greater New Orleans area, April 2021 to April 2023.

Characteristics	Summary
	*n* = 154
Age	
Median years (interquartile range)	52 (40–63)
Years of living in the USA	
Median (interquartile range)	18 (8–25)
Gender, % (*n*)	
Female	67 (103)
Male	32 (49)
Chose not to respond	1 (2)
Country of birth, % (*n*)	
Honduras	32 (50)
Nicaragua	11 (17)
USA	10 (16)
Colombia	8 (12)
Guatemala	8 (12)
Mexico	8 (12)
Ecuador	6 (9)
Peru	4 (6)
Venezuela	3 (4)
Brazil	2 (3)
Cuba	2 (3)
Panama	1 (2)
Bolivia	1 (2)
Argentina	0.6 (1)
Chile	0.6 (1)
Uruguay	0.6 (1)
El Salvador	0.6 (1)
Dominican Republic	0.6 (1)
Puerto Rico	0.6 (1)
Language participants chose for the survey, % (*n*)	
Spanish	84.2 (130)
English	15.8 (24)

**Table 2:** Awareness and knowledge of Chagas disease before and after educational intervention), in the entire sample and by gender.

Survey Questions and Responses	Before Intervention			After Intervention **		
	Overall % (*n*/N)	Female % (*n*/N)	Male % (*n*/N)	Overall % (*n*/N) **	Female % (*n*/N)	Male % (*n*/N)
* Chagas disease is caused by a						
Parasite	57 (88/154)	59 (61/103)	55.0 (27/49)	91 (134/147) **	92 (90/98)	92 (43/47)
Virus/Bacteria	4 (6/154)	3.8 (4/103)	4.0 (2/49)	3 (4/147)	3 3/98)	2 (1/47)
Don’t know/not sure	39 (60/154)	36.8 (38/103)	40.8 (20/49)	3 (/147)	2 (2/98)	2 (1/47)
No response	-	-	-	3 (5/147)	3 (3/98)	4 (2/47)
Recalled seeing triatomine insects						
Yes	52(80/154)	50 (51/103)	57 (28/49)	65 (95/147)	61 (60/98)	70 (33/47)
No	31 (47/154)	32 (33/103)	29 (14/49)	22 (33/147)	29 (28/98)	10 (5/47)
I’m not sure	16 (25/154)	17 (18/103)	12 (6/49)	12 (18/147)	10 (10/98)	17 (8/47)
No response	1 (2/154)	1 (1/103)	2 (1/49)	1 (1/147)	-	2 (1/47)
* Recognized one, two, or all three images of triatomine insects						
Yes	26 (40/154)	28 (29/103)	20 (10/49)	35 (51/147)	38 (37/98)	30 (14/47)
No	74 (114/154)	72 (74/103)	80 (39/49)	58 (85/147)	53 (52/98)	66 (31/47)
No response	-	-	-	7 (11/147)	9. (9/98)	4 (2/47)

* Statistically significant difference between before and after intervention (data for participants with pre- and post-questionnaires), McNemar’s chi-square test, *p* < 0.05. ** Only 147 participants continued with the post-intervention questionnaire. Seven participants did not complete the post-intervention questionnaire.

## Data Availability

Available on reasonable request.

## Supplementary Materials

The following supporting information can be downloaded at: https://www.mdpi.com/article/10.3390/tropicalmed8120515/s1, Table S1: Characteristics and knowledge of participants who screened positive for *T. cruzi* infection (*n* = 5).
