# Supplementary material for: Assessment of Community Awareness and Screening of Chagas Disease in the Latin American Community of Greater New Orleans
Source: Trop Med Infect Dis. 2023 Dec 5;8(12):515. doi: 10.3390/tropicalmed8120515 (PMC10747578; doi:10.3390/tropicalmed8120515)
Supplement: Supplementary file 1 [file tropicalmed-08-00515-s001.zip › tropicalmed-2713971-SI.pdf]

Supplementary Table S1. Characteristics and knowledge of participants who screened positive for T. *cruzi* infection (n=5)

	n (%)
Age categories, in years	
40-49	1 (20%)
50-59	4 (80%)
Sex	
Female	3 (60%)
Male	2 (40%)
Country of birth	
Bolivia	1 (20%)
Honduras	3 (60%)
El Salvador	1 (20%)
Years living in the US.	
<20 years	3 (60%)
>20 years	2 (40%)
Been told by a physician of their diagnosis of	
Chagas disease.	1 (20%)
Yes	4 (80%)
No	
